# Editorial

**Published:** 1898-08

**Authors:** 


### Editorial.

There are at present three hundred cases of typhoid fever under treatment at Fort McPherson. The cases were brought from Fernandina and Tampa, Florida, and Chickamauga, Georgia.

The water supply of the fort is obtained from two artesian wells, five hundred and two hundred feet deep.

It will be remembered that when this fort was first employed as a rendezvous there was almost a water famine, the supply proving entirely inadequate to the suddenly increased demand. It was the duty of the government to obtain water from the city, which could have been done at a cost inconsiderable in respect to the benefits thus conferred.

The presence of a large number of cases of enteric fever in a community supplied with well-water is a source of danger, if not immediate, at least a future menace.

It may be said that the wells being of the artesian type are not liable to contamination. It has been abundantly proven that unless the well is bored through horizontally running rock stratum, the water is not much more likely to remain wholesome when exposed to danger of contamination than that from a shallow well. If the stratum is uptilted, the well is bored through several lines of cleavage or fissures which form excellent avenues for draining surface-water into the well, however deep it may be. Where so much is at stake, it is astonishing that the government should allow a potential danger of this character to remain.

It would be best for humanity if every well in christendom were filled up and abandoned.

These cases were taken from Southern camps and have been well advertised. They have furnished a text for the newspapers of the Northwest to bitterly arraign the Southern climate and to demand the removal north of the troops exposed.

That the spread of fever is due not to climatic conditions, but to failure on the part of the officers to enforce the ordinary rules of hygiene and sanitation is shown by no less a person than Surgeon-General Sternberg. In a recent newspaper interview, he emphatically denies the charge against the healthfulness of the South, and calls attention in support of his argument to the condition of affairs at Camp Ramsey, Minnesota, Camp Alger, Virginia, and other camps of instruction in the North and West where the health of the soldiery is no better than elsewhere.

He fastens the responsibility for these unfortunate conditions upon the medical officers of the volunteer forces, chiefly because, it is presumed, he wishes to protect the officers of the regular army, for whom he must stand sponsor. It is in the highest degree probable that the volunteer medical officers are every whit as efficient and as well informed on sanitary matters as those of the regular army, for if there is superior ability among the latter it has been long and thoroughly concealed.

Sternberg calls attention to a paragraph in a circular issued by him in April, which touches the seat of the trouble:

"No doubt typhoid fever, camp diarrhea, and probably yellow fever are frequently communicated to soldiers in camp through the agency of flies, which swarm about fecal matter and filth of all kinds deposited upon the ground or in shallow pits, and directly convey infectious material, attached to their feet or contained in their excreta, to the food which is exposed while being prepared at the company kitchens or while being served in the mess tent. It is for this reason that a strict sanitary police is so important. Also, because the water supply may be contaminated in the same way, or by surface drainage."

He recommends that camps be frequently removed in order to escape the pestilential conditions which spring up about congested humanity.

A committee of investigation has been appointed to look into the conditions that have prevailed at the various camps. The commission is composed of Surgeon Major Reed, U. S. A., Victor C. Vaughn of Michigan, and E. O. Shakspere of Pennsylvania.

As usual, the South has no representative upon this commission, but it is hoped that the charges brought against her will be fully refuted.

The reproach of the general practitioner being only a skimmer upon the vast surface of medical lore seems set aside in Kansas, if one may judge by the jumbling of branches in a Kansas City institution. It speaks well for the versatility of the Kansas medico when we read that the same man is professor of dermatology and lecturer on obstetrics, while another combines diseases of the rectum with histology and microscopy; still another, hygiene, State medicine and anatomy. The chair of visceral anatomy is filled by a woman, probably a delicate compliment on the part of her colleagues paid her in deference to the prevailing ailments of her sex. There is nothing small about Kansas, except the demand for socks, and perhaps even that has gone up since Jerry Simpson has gone down.

---

A committee on the part of the alumni of the Southern Medical College conferred with a committee from the trustees of the joint institution concerning the legality of the consolidation of the Southern and the Atlanta Medical Colleges. It was contended by the first-named committee that the fusion of the two colleges was made contrary to law and could not be ratified without legislative enactment, and it requested a reconsideration of the union. The trustees' committee voted down the request, but the alumni committee expressed themselves as being dissatisfied with this action, and will test the legality of it in the courts. It appears to us that the time has passed when such action on the part of the alumni may not merit the criticism of being anachronistic.

---

Messrs. Sharp & Dohme are mailing their latest price-list of pharmaceuticals. The reliability of the products of this house is proverbial, and with their usual liberality they will be happy to send samples and copies of their lists to all readers of the RECORD who will apply to them.

---

Dr. E. Van Goidtsnoven, at a recent call meeting of the Atlanta Society of Medicine, was elected to the office of treasurer to succeed Dr. L. B. Grandy, who is now serving as surgeon of the Third Georgia regiment of infantry.

The manager of the English-American building has kindly tendered the Atlanta Medical Society the use of one of its largest rooms. The room is of ample dimensions and is well ocated. Donations of books are requested, as it is hoped that the nucleus of a library may be formed—a want keenly felt by every member of the Society. It is hoped that with a permanent home and prospect for a library, the Society will have better days before it.

---

The sympathy of this journal is extended to Dr. James B. Baird in his sad affliction in the death of his wife. She was the daughter of the late General Gartrell, and was a lady of great personal beauty and accomplishment.

---

Dr. George H. Stubbs, a graduate of the Southern Medical College of the class of '96, has been appointed to the position of oculist and aurist to the Southern Railway, west of Atlanta. Dr. Stubbs resides at Birmingham.

---

Dr. T. C. Longino has accepted a position as contract surgeon in the Department of the Gulf, and is now stationed at Galveston, Texas.

---

Dr. Henry Bok, formerly of Atlanta and late of Chicago, has returned to this city.

